# Effects of Dietary Crude Protein Level on Growth Performance, Immune Function, and Serum Metabolic Profile in Weaned Piglets

**DOI:** 10.3390/ani16162464

**Published:** 2026-08-08

**Authors:** Tao Wang, Jin Chen, Siyu Xiong, Xiurong Peng, Jinyuan Liu, Dong Qu, Bin Li, Jiabao Yang

**Affiliations:** 1 Sichuan Animal Science Academy, Chengdu 610066, China; 2 Animal Genetic Breeding and Reproduction Key Laboratory of Sichuan Province, Chengdu 610066, China

**Keywords:** weaned piglets, crude protein, growth performance, diarrhea, immunoglobulin, serum metabolomics

## Abstract

Dietary crude protein (CP) level is a critical nutritional factor influencing growth and intestinal health in weaned piglets. This study investigated the effects of graded dietary CP levels on growth performance, diarrhea incidence, serum immune indices, and metabolic profiles in weaned piglets. Five dietary CP levels (14%, 15%, 16%, 17%, and 18%) were tested, and growth performance and diarrhea incidence were recorded during a 14-day trial. Representative piglets from the 14%, 16%, and 18% CP groups were subsequently selected for serum biochemical and immunological analyses, as well as LC-MS-based untargeted metabolomics. The results showed that average daily gain (ADG) increased linearly (*p* = 0.004) and the feed-to-gain ratio (F/G) decreased linearly (*p* = 0.001) with increasing dietary CP level. However, diarrhea rate exhibited a quadratic trend in response to dietary CP level, with the lowest value observed for the 16% CP group, indicating a potential optimal intestinal-health response at this CP level. Serum total protein (TP) and immunoglobulins (IgA, IgG, and IgM) were significantly higher in the 18% CP group than in the 14% CP group (*p* < 0.05). Metabolomic analysis revealed clear separation among the 14% (*n* = 5), 16% (*n* = 4), and 18% (*n* = 4) CP groups in the PLS-DA model. Differential metabolites were mainly enriched in lipid and amino acid metabolic pathways, with amino acid metabolism being most strongly affected, involving several dipeptides and amino acid derivatives (e.g., Tyr-Pro, His-Leu, and Phe-Trp). Collectively, increasing dietary CP linearly improved growth performance and humoral immunity, whereas diarrhea incidence showed a quadratic numerical trend with the lowest value at 16% CP. These findings provide new metabolic insights for optimizing dietary CP formulation in weaned piglets.

## Simple Summary

Weaning stress in piglets often results in retarded growth and diarrhea. Dietary protein level is of critical importance: insufficient protein suppresses growth and impairs immunity, whereas excessive protein readily induces diarrhea. In this study, weaned piglets were fed diets containing five different protein levels to evaluate the effects of dietary protein on growth, diarrhea, blood immune indicators, and the serum metabolic profile. The results showed that increasing the dietary protein level improved the growth performance and blood immune indicators of piglets. Metabolomic analysis further revealed that different protein levels reshaped the serum metabolic profile of weaned piglets. These findings help to determine an appropriate dietary protein level that balances growth performance with intestinal health.

## 1. Introduction

Weaning is one of the most critical phases in swine production. During this period, piglets face nutritional and environmental stressors associated with the transition from sow milk to solid feed, which frequently results in decreased feed intake, impaired intestinal barrier function, a high incidence of diarrhea, and growth retardation [1,2]. As an indispensable nutrient for growth and immune development, dietary protein directly determines the health status and productive performance of weaned piglets [3].

The optimal dietary crude protein (CP) level for weaned piglets has been a longstanding focus in animal nutrition. Traditional feeding strategies have favored high-protein diets (typically >20%) to meet the amino acid requirements for rapid growth [3,4,5]. However, because the digestive system of piglets is not yet fully mature, excess dietary protein cannot be completely digested and absorbed in the small intestine. The undigested protein reaches the hindgut, where it is fermented by pathogenic microorganisms into toxic metabolites such as ammonia, amines, and hydrogen sulfide, thereby disrupting the intestinal microecology and predisposing piglets to nutritional diarrhea [6,7]. To alleviate this problem and to reduce nitrogen emissions, low-protein amino acid-balanced diets have been widely adopted. In modern commercial swine production, low-protein diets are generally formulated by supplementing crystalline indispensable amino acids (L-lysine, DL-methionine, L-threonine and L-tryptophan) to meet the essential amino acid requirements of piglets. This strategy reduces dietary crude protein by 2–4 percentage points, and can effectively lower diarrhea incidence and decrease nitrogen excretion [8]. However, excessive reduction in dietary crude protein (e.g., below 16%), even with the supplementation of essential amino acids, frequently results in insufficient provision of non-essential functional amino acids such as glutamine and arginine in weaned piglets. This deficiency impairs immunoglobulin synthesis and immune function development, ultimately compromising overall growth performance [9,10]. Therefore, under continuous low-protein gradient conditions, the precise determination of the optimal dietary crude protein level that simultaneously guarantees growth performance, immune function, and intestinal health remains an urgent issue to be addressed through systematic dose–response studies. In addition, advances in high-throughput analytical technologies have made metabolomics a powerful tool for exploring the underlying mechanisms of nutritional regulation in livestock. Different dietary protein intakes inevitably redistribute the circulating metabolite pool [11,12]. Previous studies have largely focused on free amino acid concentrations, while the roles of small peptides (e.g., di- and tripeptides) and lipid intermediates in mediating the interaction between dietary nutrients and host physiology have received little attention.

Currently, commercial diets for 7–20 kg weaned piglets in China generally contain 18.0–20.0% CP under the traditional high-protein feeding mode. With the popularization of low-protein amino acid-balanced technology, most farms have reduced dietary CP to 15.0–17.0% in practice, while further reduction to below 14.0% is rarely applied due to concerns about growth restriction. Therefore, we set a continuous CP gradient from 14% to 18% to cover the main application range of commercial low-protein diets and to accurately identify the dose–response relationship between CP level and physiological parameters. Accordingly, the present study established a continuous gradient of dietary CP (14–18%) to systematically evaluate its linear and non-linear effects on growth performance, diarrhea incidence, and serum biochemical and immunological parameters in weaned piglets. We hypothesized that: (1) growth performance would increase linearly with dietary CP level within this range, while diarrhea incidence would show a quadratic response with the lowest value at a moderate CP level, and (2) dietary CP level would systematically reshape the serum metabolome, which would be associated with changes in growth and immune phenotypes. In parallel, LC-MS-based untargeted metabolomics was used to characterize differences in serum metabolic profiles among representative CP levels, aiming to elucidate the metabolic mechanisms by which dietary protein modulates growth and health, thereby providing a theoretical basis for the precise application of low-protein diets in swine production.

## 2. Materials and Methods

### 2.1. Ethics Statement

The management of animal experiments involved in the research refers to the “Regulations on the Administration of Laboratory Animals” (Ministry of Science and Technology, China, revised in June 2004). The sample collection was approved by the Sichuan Animal Science Academy, Sichuan, China (No. 2026017).

### 2.2. Experimental Design

A total of 130 healthy weaned barrows (initial body weight, 7.34 ± 0.12 kg) were assigned to five dietary treatments in a stratified randomized design, in which piglets were first stratified by body weight and then randomly allocated to treatments within each stratum. The treatments provided 14%, 15%, 16%, 17%, or 18% crude protein (CP), with five piglets per pen and six replicate pens for the 14% CP treatment and five replicate pens for each of the remaining treatments. The extra replicate in the 14% CP treatment was included because surplus eligible piglets with matched initial body weights were available at the time of allocation. This slightly unbalanced design modestly improves the precision of the estimates for the low-protein treatment without inflating the Type I error rate of the one-way ANOVA, as ANOVA is robust to moderate inequality in sample sizes when the assumption of homogeneity of variance is satisfied.

### 2.3. Animal Housing and Management

The trial was conducted at our Swine Nutrition Experimental Base in Lezhi County, Ziyang City, Sichuan Province, China, and lasted for 14 days. Piglets had ad libitum access to feed and water. Routine husbandry and immunization procedures followed the Base’s standard operating procedures. Feed intake, health status, and fecal consistency were recorded daily. Piglets were housed in fully slatted floor pens (2.0 m × 3.0 m) with 5 piglets per pen, resulting in a housing density of 1.2 m^2^ per piglet. The house was equipped with automatic tunnel ventilation. The ambient temperature was maintained at 26–28 °C during the first week and 24–26 °C during the second week, with relative humidity controlled at 55–65%. Lighting was provided 12 h per day (07:00–19:00). The basal diet was corn–soybean meal-based, and the ingredient composition and calculated nutrient levels are presented in Table 1.

### 2.4. Sample Collection

On the morning of day 14 (06:00), one piglet was randomly selected from each replicate, and 5 mL of blood was collected from the anterior vena cava. Serum was separated by centrifugation at 2000× *g* for 3 min and stored at −20 °C until analysis.

### 2.5. Measurements

#### 2.5.1. Growth Performance

Piglets were weighed individually after an overnight fast at the beginning and end of the trial, and feed intake was recorded weekly per replicate. Average daily gain (ADG), average daily feed intake (ADFI), and feed-to-gain ratio (F/G) were then calculated.

#### 2.5.2. Diarrhea Incidence

Fecal consistency was scored three times daily (morning, noon, and evening) as follows: 1 = soft, partially formed feces; 2 = pasty feces without separation of water; and 3 = watery feces with separation of water. Diarrhea frequency and diarrhea index were calculated as:

Diarrhea frequency (%) = (total number of diarrheic piglets)/(number of piglets × days) × 100;

Diarrhea index = sum of diarrhea scores/number of piglets.

#### 2.5.3. Serum Biochemical and Immunological Indices

Serum concentrations of immunoglobulin A (IgA), immunoglobulin G (IgG), immunoglobulin M (IgM), total protein (TP), albumin (ALB), and blood urea nitrogen (BUN) were quantified using commercial kits from Nanjing Jiancheng Bioengineering Institute (Nanjing, China; catalogue Nos. H108-1-2, H106-1-1, H109-1-2, A045-2-1, A028-2-1, and C013-2-1, respectively), following the manufacturer’s protocols.

#### 2.5.4. Untargeted Metabolomics Analysis

Sample preparation. Serum (100 μL) was pipetted into a 1.5-mL centrifuge tube together with an equal volume of acetonitrile/water (1:1, *v*/*v*) spiked with four internal standards, one of which was L-2-chlorophenylalanine, to allow for data correction. The mixture was vortexed (30 s), ultrasonicated at 40 kHz and 5 °C for 30 min, and left at −20 °C for a further 30 min to precipitate proteins. Following centrifugation (13,000× *g*, 4 °C, 15 min), the resulting supernatant was evaporated to dryness under a nitrogen stream and redissolved in 120 μL of the same solvent. A second round of vortexing (30 s), brief sonication (5 °C, 5 min), and centrifugation (13,000× *g*, 4 °C, 10 min) yielded the final extract, which was transferred to autosampler vials for LC-MS analysis.

Quality control. Quality control (QC) samples were prepared by pooling 20 μL of supernatant from each serum sample and were injected in parallel with the study samples to monitor instrument stability. Peak areas of all metabolites were normalized to that of L-2-chlorophenylalanine to correct for injection variation and matrix effects. Across all samples, the relative recovery of L-2-chlorophenylalanine ranged from 92.3% to 107.8%, and the coefficient of variation (CV) of its peak area across QC injections was 4.7%, indicating acceptable recovery and good analytical stability.

UHPLC conditions. Chromatographic separation was performed on a Thermo Vanquish Horizon UHPLC system equipped with an ACQUITY UPLC HSS T3 column (100 mm × 2.1 mm, 1.8 μm; Waters, Milford, MA, USA). The column oven temperature was set at 40 °C, the flow rate was 0.3 mL/min, and the injection volume was 3 μL. Mobile phase A consisted of 95% water and 5% acetonitrile with 0.1% formic acid; mobile phase B consisted of 47.5% acetonitrile, 47.5% isopropanol, and 5% water with 0.1% formic acid. The gradient elution program was as follows: 0–2 min, 2% B; 2–11 min, 2–50% B; 11–14 min, 50–95% B; 14–16 min, 95% B; 16–16.1 min, 95–2% B; 16.1–20 min, 2% B for column re-equilibration.

Mass spectrometry conditions. Detection was carried out on a Thermo Exploris 480 mass spectrometer (Thermo Scientific, Waltham, MA, USA) with electrospray ionization (ESI) operating in both positive and negative modes over an *m*/*z* range of 70–1050. The main parameters were: sheath gas flow rate, 50 arb; auxiliary gas flow rate, 15 arb; ion transfer tube temperature, 400 °C; capillary temperature, 350 °C; spray voltage, +3400 V (positive) and −2800 V (negative); S-Lens RF voltage, 50; normalized collision energies, 20%, 40%, and 60%; MS1 resolution, 60,000; MS2 resolution, 7500.

Quality control during acquisition. One QC sample was injected after every 5–15 experimental samples. Instrument stability and reproducibility were evaluated using the total ion chromatograms (TICs) and internal standard *z*-score distributions of QC samples, with ±2 SD used as the acceptance threshold. All internal standard *z*-scores fell within −2 to +2, confirming the stability of the analytical system.

Data processing and metabolite identification. Raw data were imported into Progenesis QI v3.0 for baseline filtering, peak detection, integration, retention time correction, and peak alignment, generating a standardized data matrix containing retention time, *m*/*z*, and peak area. Metabolites were identified by matching against the HMDB, METLIN, and an in-house database (Majorbio), with a mass tolerance of 10 ppm at the MS1 level combined with MS2 fragment matching scores. Annotation information included KEGG Compound ID, molecular formula, adduct ions, CAS number, and database matching score.

### 2.6. Statistical Analysis

Data were preliminarily organized in Microsoft Excel 2007 and analyzed using SPSS 18.0. Prior to analysis, all data were tested for normality using the Shapiro–Wilk test and for homogeneity of variance using Levene’s test. All parameters met the assumptions of normality and equal variance, and parametric analyses were therefore applied. One-way analysis of variance (ANOVA) was used to compare differences among the five dietary treatment groups, followed by Tukey’s HSD post-hoc test. Chi-square test was used for the analysis of categorical variables when appropriate. Orthogonal polynomial contrasts were further applied to test for linear and quadratic trends across the dietary protein levels. KEGG pathway enrichment analysis of differential metabolites was performed using the hypergeometric distribution test, and *p*-values were adjusted by false discovery rate (FDR) correction for multiple hypothesis testing. Pathways with FDR-adjusted *p*-value < 0.05 were considered significantly enriched. All results are reported as means ± SEM, with *p* < 0.05 and *p* < 0.01 taken to indicate significant and highly significant differences, respectively.

## 3. Results

### 3.1. Effects of Dietary CP Level on Growth Performance

As shown in Table 2, dietary CP levels had no significant effect on piglet body weight on day 7 or day 14 (*p* > 0.05). With respect to average daily gain (ADG), alterations in dietary CP levels significantly affected the ADG of piglets during both the 0–7 d and the overall 0–14 d periods (*p* < 0.05). During days 0–7, the ADG of piglets in the 16% and 18% CP groups was significantly higher than that of the 14% CP group (*p* < 0.05), and the ADG of the 18% CP group was significantly higher than that of the 15% CP group (*p* < 0.05). Over the entire 0–14 d period, the ADG of the 16% and 18% CP groups was significantly higher than that of the 14% low-protein group (*p* < 0.05), and a highly significant linear increase was observed with increasing dietary CP levels (*p* < 0.05). No significant differences in average daily feed intake (ADFI) were detected among treatments at any stage (*p* > 0.05). Regarding the feed-to-gain ratio (F/G), increasing dietary CP levels significantly improved piglet feed efficiency. During days 0–7, the F/G of the 18% CP group was significantly lower than that of the 14% and 15% CP groups (*p* < 0.05). Over the entire 0–14 d period, the F/G values of the 16%, 17%, and 18% CP groups were significantly lower than those of the 14% and 15% CP groups (*p* < 0.05). Furthermore, regression analysis revealed that F/G decreased linearly and significantly with increasing dietary CP levels during both the 0–7 d and the overall 0–14 d periods (*p* < 0.05).

### 3.2. Effects of Dietary CP Level on Diarrhea Rate and Diarrhea Index

Effects of dietary CP on diarrhea in weaned piglets are shown in Table 3. ANOVA revealed no significant differences among treatments in diarrhea rate or diarrhea index during days 0–7, 8–14, or 0–14 (*p* > 0.05). However, polynomial contrast analysis showed that intestinal health responded to dietary CP in a non-linear manner. During days 0–7, diarrhea rate tended to follow a quadratic pattern (quadratic *p* = 0.06), decreasing from 11.43% in the 14% CP group to a minimum of 7.62% in the 16% CP group and rebounding to 12.26% in the 18% CP group. Similar quadratic patterns, although not statistically significant, were observed for diarrhea rate over days 0–14 and for the diarrhea index across all periods. Numerically, piglets in the 16% CP group consistently showed the lowest diarrhea frequency and severity, indicating a potential trend toward improved intestinal health at this CP level, although the difference did not reach statistical significance.

### 3.3. Effects of Dietary CP Level on Serum Biochemistry and Immunity

**Figure 1:**
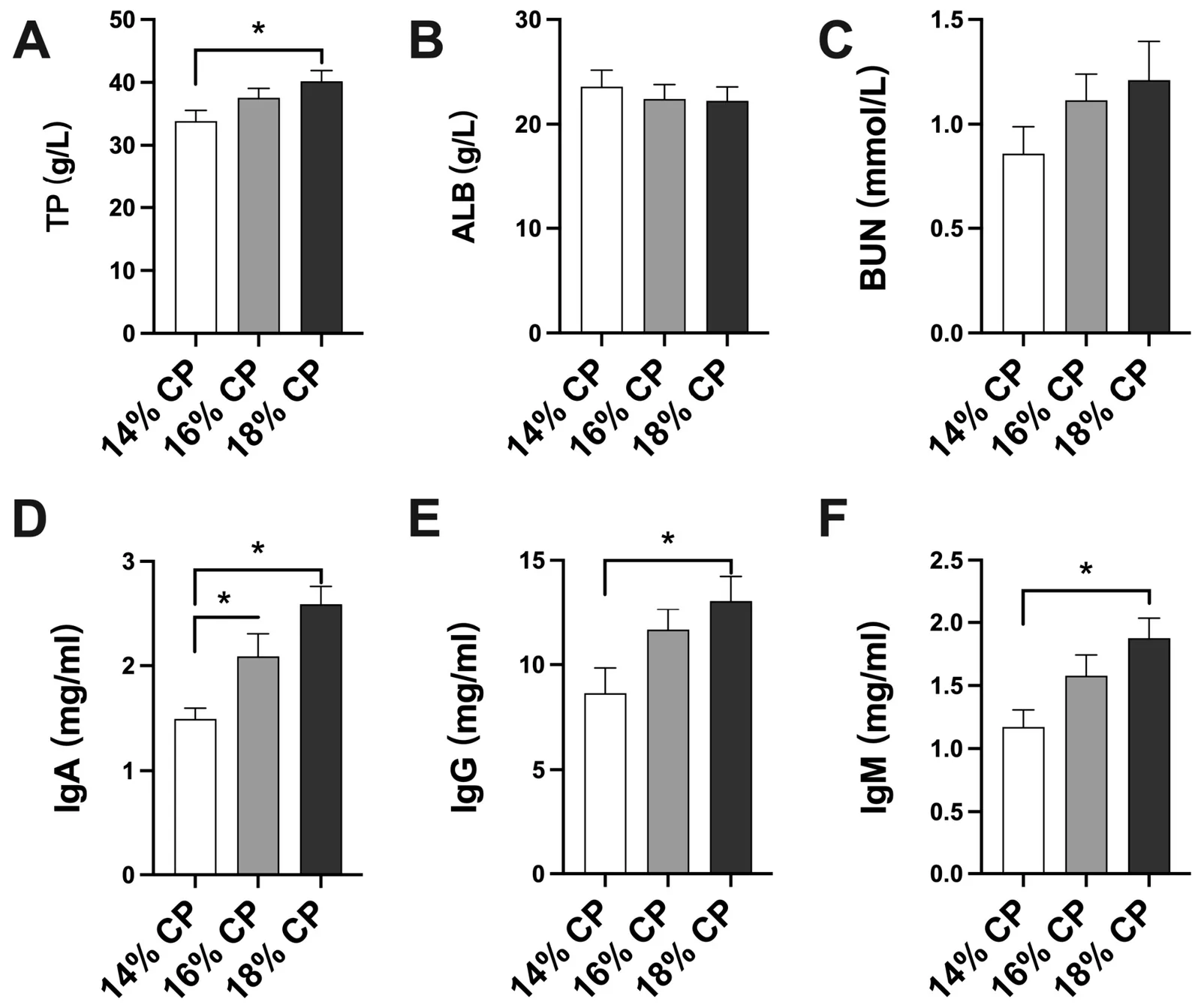
Effects of dietary crude protein levels on serum biochemical and immune parameters in weaned piglets. Serum concentrations of total protein (TP, (**A**)), albumin (ALB, (**B**)), blood urea nitrogen (BUN, (**C**)), immunoglobulin A (IgA, (**D**)), immunoglobulin G (IgG, (**E**)), and immunoglobulin M (IgM, (**F**)). Data are presented as means ± SEM (*n* = 5–6 per group). Asterisks (*) indicate significant differences (*p* < 0.05).

Based on growth performance and diarrhea outcomes, piglets from the 14%, 16%, and 18% CP groups were selected as representative groups for serum analyses. Serum TP responded to dietary CP level, with the 18% CP group exceeding the 14% CP group (*p* < 0.05). In contrast, neither ALB nor BUN was influenced by treatment (*p* > 0.05). With respect to humoral immunity, serum concentrations of IgA, IgG, and IgM increased with dietary CP level. Specifically, IgA, IgG, and IgM in the 18% CP group were significantly higher than in the 14% CP group (*p* < 0.05), and serum IgA in the 16% CP group was also significantly higher than in the 14% CP group (*p* < 0.05) (Figure 1).

### 3.4. Effects of Dietary CP Level on Serum Metabolic Profile

**Figure 2:**
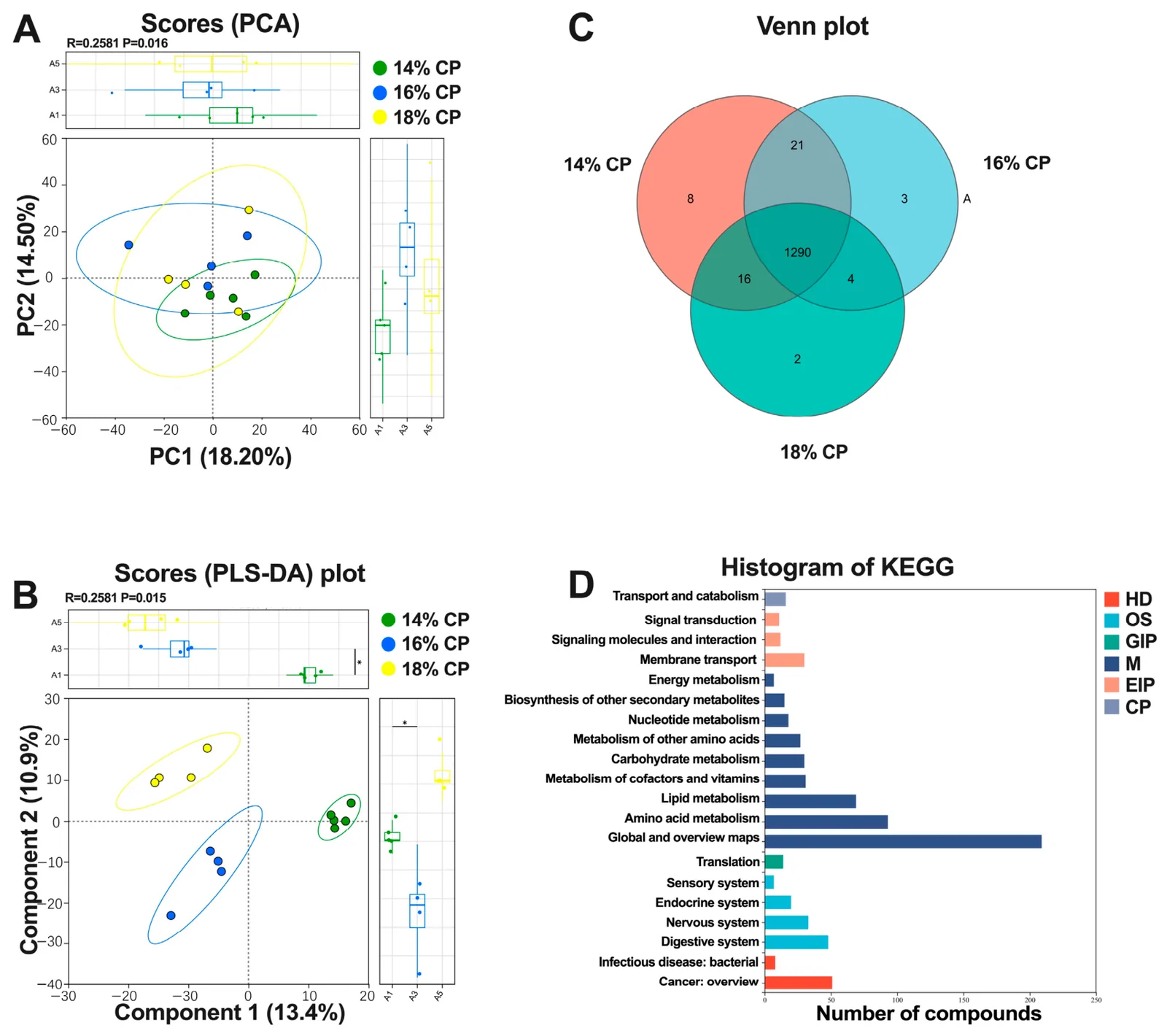
Effects of different dietary crude protein (CP) levels on the serum metabolomic profiles of weaned piglets. (**A**) Principal component analysis (PCA) score plot showing the distribution of serum metabolites among the 14% CP, 16% CP, and 18% CP groups. (**B**) Partial least squares discriminant analysis (PLS-DA) score plot (R^2^X = 0.353, R^2^Y = 0.982, Q^2^ = 0.540). (**C**) Venn diagram illustrating the numbers of shared and unique serum metabolites among the 14% CP, 16% CP, and 18% CP groups. (**D**) KEGG pathway annotation histogram of the identified serum metabolites, in which the vertical axis lists the functional categories and the horizontal axis represents the number of annotated compounds (*n* = 4–5 per group).

**Figure 3:**
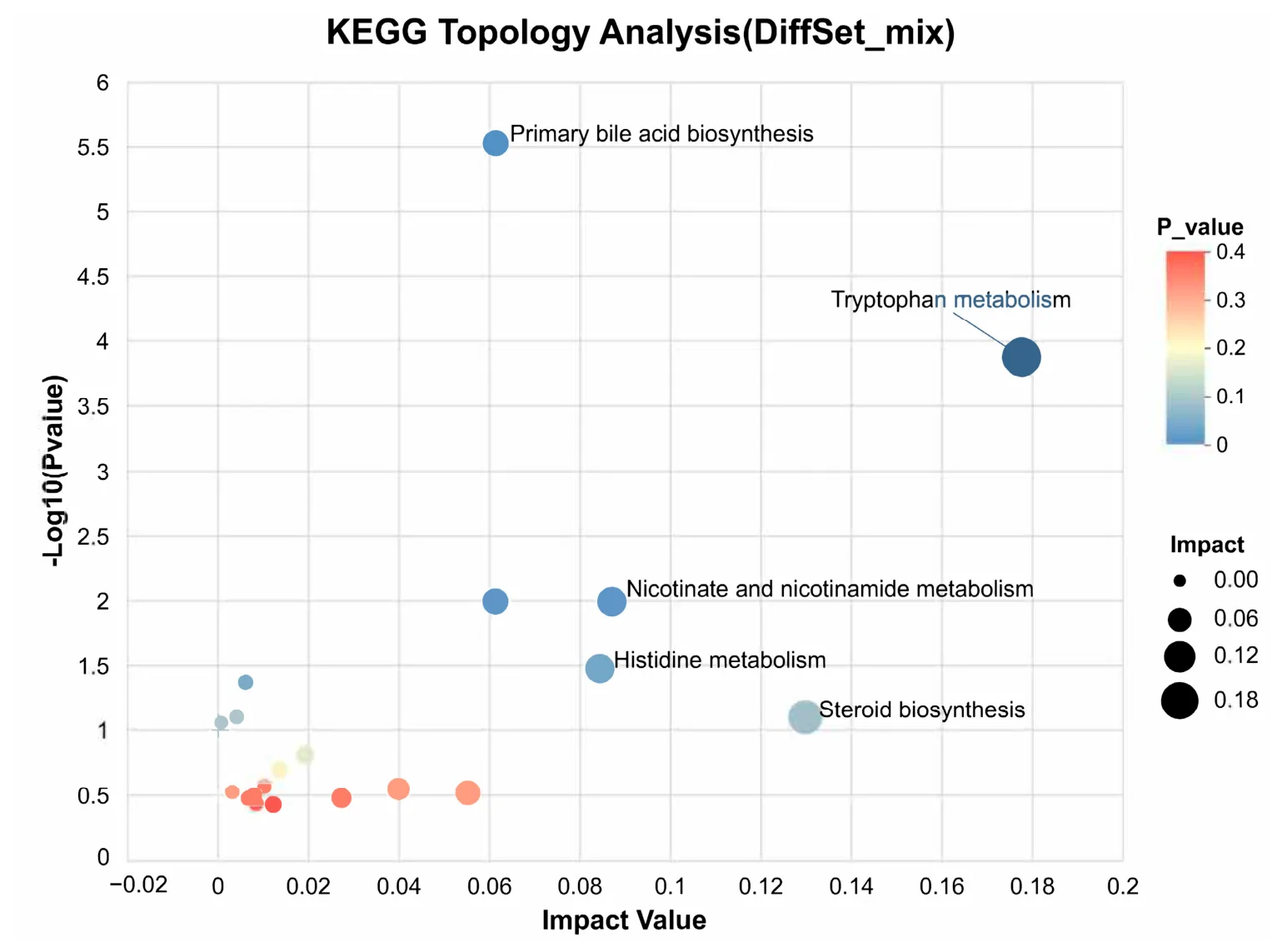
KEGG pathway topology analysis of differential serum metabolites among piglets fed different dietary crude protein levels. Bubble plot displaying the KEGG pathway enrichment and topology analysis of differential metabolites (DiffSet_mix) identified among the 14% CP, 16% CP, and 18% CP groups. The horizontal axis represents the pathway impact value derived from topology analysis (higher values indicate greater importance of the pathway within the metabolic network), and the vertical axis represents the enrichment significance expressed as −log_10_ (*p*-value) (*n* = 4–5 per group).

**Figure 4:**
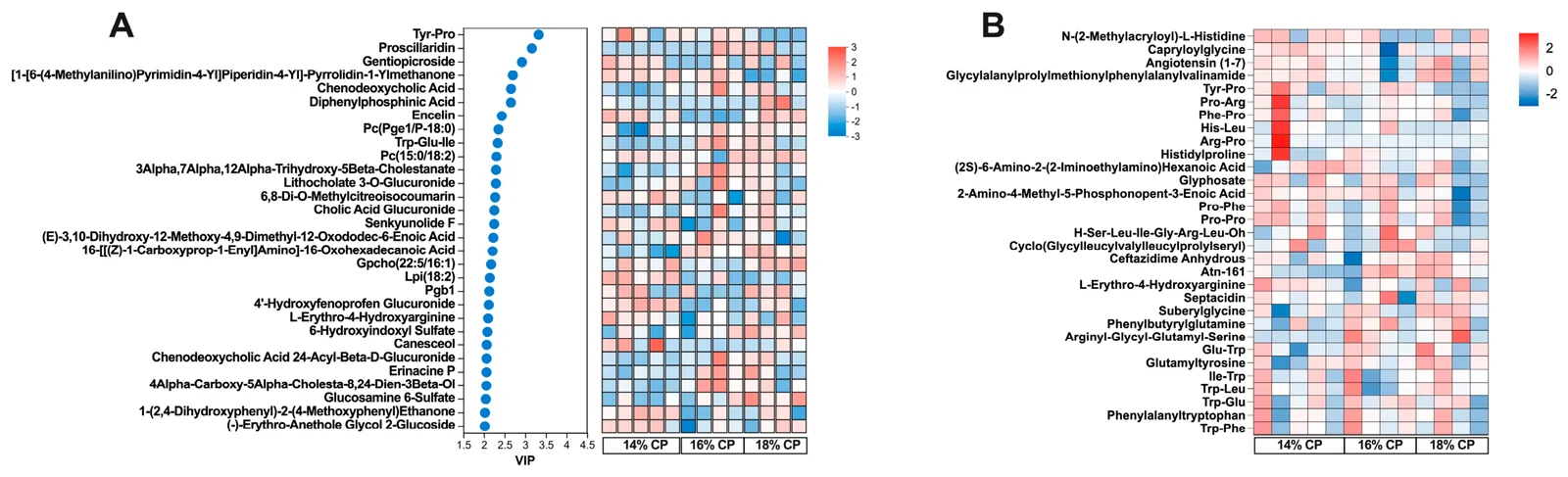
Serum metabolomic profiles of weaned piglets fed diets with different crude protein levels. (**A**) Variable importance in projection (VIP) scores (left) of the top differential metabolites (VIP > 1.5) identified by PLS-DA, alongside a heatmap (right) illustrating their relative abundances across the 14% CP, 16% CP, and 18% CP groups. (**B**) Heatmap of differentially abundant peptides and small-molecule metabolites (mainly amino acid-related dipeptides and derivatives, e.g., Tyr-Pro, Trp-Glu, Trp-Phe, Phe-Pro, His-Leu) among the three dietary groups (*n* = 4–5 per group).

To further elucidate the regulatory effects of dietary CP on the serum metabolome, untargeted metabolomics was performed. Principal component analysis (PCA) revealed partial separation among the 14%, 16%, and 18% CP groups (Figure 2A). Partial least squares discriminant analysis (PLS-DA) yielded distinct clustering of samples within each group and clear discrimination between the three dietary treatments in both ESI+ and ESI− modes (Figure 2B and Supplementary Figure S1). The partial separation in PCA reflects the overall metabolic variation among individuals, while the clearer separation in the supervised PLS-DA model indicates that dietary CP level is an important source of metabolic variation. The reliability of the PLS-DA model was verified by permutation testing, which showed no evidence of overfitting. Venn analysis identified 8, 3, and 2 unique metabolites in the 14%, 16%, and 18% CP groups, respectively (Figure 2C and Table S1). Kyoto Encyclopedia of Genes and Genomes (KEGG) annotation showed that the identified metabolites were mainly enriched in lipid and amino acid metabolism (Figure 2D), suggesting that dietary CP primarily modulates lipid and amino acid metabolic processes in piglets. Pathway enrichment analysis of differential metabolites confirmed that amino acid metabolism was the most strongly affected pathway (Figure 3). Key differential metabolites involved in this pathway included Tyr-Pro, hydroxyindoxyl sulfate, N-(2-methylacryloyl)-L-histidine, His-Leu, His-Pro, Glu-Trp, Ile-Trp, Trp-Leu, Trp-Glu, and Phe-Trp (Figure 4A,B and Table S2).

## 4. Discussion

This study evaluated the effects of graded dietary CP levels on growth performance, immune function, and serum metabolic profiles in weaned piglets. Growth performance (ADG and F/G) improved linearly with increasing dietary CP, and serum immunoglobulin concentrations rose accordingly. In contrast, diarrhea incidence exhibited a clear quadratic dose–response, with the 16% CP group showing the best intestinal health. Furthermore, untargeted metabolomics revealed that dietary CP substantially reshaped serum lipid and amino acid metabolic networks, particularly altering the abundance of several small peptide-containing metabolites. These findings not only support the identification of an appropriate low-protein level for weaned piglets but also provide new insights into the metabolic mechanisms by which dietary protein modulates host physiology.

Dietary protein is fundamental for muscle deposition and tissue development in piglets [13]. In the present study, increasing dietary CP from 14% to 18% significantly improved ADG during days 0–14, and F/G decreased linearly with increasing CP. These results suggest that an 18% CP level more adequately meets the amino acid requirements of early-weaned piglets and thereby improves feed efficiency. This pattern is consistent with a previous meta-analysis reporting that F/G in nursery pigs is optimized at approximately 18.4% dietary CP [14]. Notably, although ADG and F/G differed significantly, final body weights at days 7 and 14 did not differ significantly, likely because the short trial duration (14 days) was insufficient for cumulative differences in body weight to emerge. This suggests that ADG and F/G are more sensitive than final body weight for evaluating nutritional responses of weaned piglets in short-term trials.

Weaned piglets are highly susceptible to diarrhea induced by nutritional and environmental stress [15,16,17]. In the present study, although diarrhea rate and diarrhea index did not differ significantly among treatments (*p* > 0.05), polynomial regression revealed a clear quadratic response to dietary CP, with the lowest diarrhea incidence at 16% CP and a rebound at both 14% and 18% CP. This finding is consistent with previous reports indicating that excessive dietary CP exceeds the digestive capacity of weaned piglets, allowing undigested protein to reach the hindgut, where microbial fermentation produces toxic metabolites such as ammonia, amines, and hydrogen sulfide, thereby disrupting the intestinal microbiota and inducing nutritional diarrhea [18]. On the other hand, diarrhea also increased at low CP levels; when CP was reduced excessively (≤14%), insufficient supply of functional dispensable amino acids may have compromised mucosal repair and local immune barrier function. This observation aligns with the review of Heo et al., which highlighted the indispensable role of functional amino acids in maintaining small intestinal barrier and immune function [19].

Serum biochemical indices reflect the protein metabolic status of the host. In this study, serum TP was significantly higher in the 18% CP group than in the 14% CP group, indicating that a higher dietary CP level promoted body protein synthesis, in agreement with the metabolic and proteomic responses reported by Li et al. [12] in long-term protein-restricted pigs. However, serum BUN exhibited no significant differences among treatments, which differs from a previous study where low-protein diets were found to significantly decrease BUN [20]. The steady BUN concentration in the current trial suggests that under the amino acid-balanced feeding conditions, amino acid catabolism was maintained at a relatively stable compensatory level with no marked excessive deamination, indirectly reflecting that the experimental diets possessed favorable rationality regarding amino acid utilization efficiency.

With regard to humoral immunity, IgA, IgG, and IgM all increased significantly with dietary CP. Immunoglobulin synthesis depends heavily on an adequate supply of amino acids; the higher immunoglobulin levels in the 18% CP group indicate that sufficient dietary protein supports higher circulating immunoglobulin concentrations, reflecting an enhanced humoral immune capacity; however, whether this translates into improved disease resistance requires further validation via pathogen challenge experiments, consistent with previous reports on the immunomodulatory role of amino acid supply in piglets [21,22]. Importantly, serum IgA in the 16% CP group was also significantly higher than in the 14% CP group, which is in line with the lowest diarrhea rate observed in that group.

Metabolomic analysis revealed the molecular basis underlying these macro-phenotypic responses to dietary CP. PCA and PLS-DA showed that serum metabolic profiles were markedly reshaped among the 14%, 16%, and 18% CP groups, with differential metabolites enriched primarily in lipid and amino acid metabolism, the latter being the most strongly affected. These observations are consistent with previous studies on protein-restricted pig models, which reported systematic redistribution of the circulating metabolite pool in response to dietary protein manipulation [23,24]. Notably, we identified multiple dipeptides and amino acid derivatives (e.g., Tyr-Pro, His-Leu, His-Pro, Phe-Trp, and Trp-Leu) that responded to dietary CP. This finding extends previous research paradigms centered on free amino acids and highlights the potential role of small peptides in mediating the interaction between dietary nutrition and host physiology. Specifically, dipeptides such as Tyr-Pro, His-Leu, and Phe-Trp identified in this study can be efficiently transported across the intestinal epithelium via the oligopeptide transporter PepT1 [25]. Beyond serving as amino acid carriers, these dipeptides may exert bioactive functions: for example, Tyr-Pro has been reported to exhibit anti-inflammatory activity by inhibiting pro-inflammatory cytokine secretion [26]. Mechanistically, di- and tripeptides are not only more efficient carriers of amino acid absorption via PepT1, requiring less energy than free amino acid uptake, but may also act as bioactive peptides involved in receptor signaling, antioxidant defense, and immunomodulation [27,28,29]. It should be noted that the signaling and immunomodulatory roles of these dipeptides are hypothesized based on previous studies and have not been directly verified in the present experiment; their specific physiological functions in piglets require further targeted validation.

Several limitations of this study should be acknowledged. First, the 14-day trial duration only covered the acute post-weaning stress phase (0–14 days post-weaning), which cannot reflect the long-term cumulative effects of dietary CP levels on growth performance, intestinal development, and immune function throughout the entire nursery phase (e.g., 0–42 days post-weaning). The potential compensatory growth effects and persistent impacts on intestinal health during the growing–finishing period also remain unclear. Future studies extending the feeding period to the full nursery phase are needed to verify the long-term application value of low-protein diets. Second, this study only measured serum immunoglobulin levels and did not evaluate intestinal morphological structure, inflammatory cytokine profiles, intestinal barrier function biomarkers (such as diamine oxidase and D-lactic acid), or gut microbiota composition. These measurements would help to more directly elucidate the mechanisms by which dietary CP affects intestinal health and should be included in future studies. Third, although serum metabolomics revealed pronounced changes in small peptide and lipid metabolism, the specific physiological functions of individual dipeptides (e.g., Tyr-Pro) were not directly validated. Future studies integrating gut microbiome and transcriptome analyses could further clarify the causal links between hindgut fermentation and host metabolic changes. In vitro models such as IPEC-J2 intestinal epithelial cells and immune cell co-culture systems could also be employed to investigate the molecular mechanisms by which specific small peptides modulate intestinal barrier integrity and immune function, thereby providing a foundation for the precise nutritional application of functional small peptides.

## 5. Conclusions

In conclusion, increasing dietary CP from 14% to 18% linearly improved growth performance and humoral immunity in weaned piglets. Diarrhea incidence was numerically lowest at 16% CP, but the differences among groups were not statistically significant. Serum metabolomics revealed that dietary CP substantially reshaped amino acid and lipid metabolism, with multiple dipeptides (e.g., Tyr-Pro, His-Leu, and Phe-Trp) identified as key differential metabolites. Overall, these results suggest that growth performance and humoral immunity respond primarily to increasing dietary CP, whereas small peptide metabolism may represent a novel mechanistic link between dietary protein and host physiology in early-weaned piglets.

## Figures and Tables

**Table 1:** Composition and nutrient levels of the experimental diets (air-dry basis, %).

Ingredients	CP 14%	CP 15%	CP 16%	CP 17%	CP 18%
Corn (CP 8%)	53.00	50.20	47.60	44.80	42.00
Fermented soybean meal	3.00	3.00	3.00	3.00	3.00
Soybean meal (CP 43%)	5.80	8.60	11.20	14.00	16.80
Extruded full-fat soybean	6.00	6.00	6.00	6.00	6.00
Super steam-processed fish meal	4.00	4.00	4.00	4.00	4.00
Whey powder (low protein)	15.00	15.00	15.00	15.00	15.00
Glucose	8.00	8.00	8.00	8.00	8.00
Calcium formate	0.30	0.30	0.30	0.30	0.30
Dicalcium phosphate	0.90	0.86	0.82	0.78	0.74
Sodium chloride	0.20	0.20	0.20	0.20	0.20
Premix ^1^	3.80	3.84	3.88	3.92	3.96
Total	100.00	100.00	100.00	100.00	100.00
Nutrient composition					
Digestible energy (kcal/kg)	3400.00	3400.00	3400.00	3400.00	3400.00
Crude protein (%)	14.00	15.00	16.00	17.00	18.00
Calcium (%)	0.66	0.66	0.66	0.66	0.66
Total phosphorus (%)	0.60	0.60	0.60	0.60	0.60
Available phosphorus (%)	0.45	0.45	0.45	0.45	0.45
Digestible Lys (%)	0.96	1.03	1.10	1.17	1.24
Digestible Met (%)	0.36	0.39	0.42	0.45	0.48
Digestible Met + Cys (%)	0.54	0.58	0.62	0.66	0.70
Digestible Thr (%)	0.59	0.64	0.68	0.72	0.77
Digestible Trp (%)	0.18	0.20	0.21	0.22	0.24

Note: ^1^ The premix contained sodium chloride, L-lysine hydrochloride, DL-methionine, L-threonine, L-tryptophan, trace minerals, vitamins, phytase, and a compound acidifier, providing the following per kilogram of complete diet: Cu, 110 mg; Fe, 100 mg; Mn, 25 mg; Zn, 100 mg; Se, 0.3 mg; I, 0.6 mg; vitamin A, 13,000 IU; vitamin D_3_, 3500 IU; vitamin E, 30 mg; vitamin K_3_, 4.8 mg; vitamin B_12_, 0.048 mg; vitamin B_1_, 4 mg; vitamin B_2_, 11 mg; vitamin B_6_, 5.2 mg; nicotinamide, 40 mg; pantothenic acid, 21 mg; folic acid, 2 mg; biotin, 0.2 mg; phytase, 1500 U.

**Table 2:** The Effect of *Dietary CP* Level on the Growth Performance of Weaned Piglets.

Items	CP Level, %					SEM	*p*-Value		
	14%	15%	16%	17%	18%		ANOVA	Linear	Quadratic
Body Weight, kg									
0 d	7.36	7.34	7.30	7.34	7.36	0.12	1.000	0.988	0.999
7 d	8.41	8.46	8.78	8.47	8.96	0.15	0.721	0.300	0.847
14 d	10.17	10.46	10.88	10.62	11.19	0.18	0.423	0.088	0.939
ADG, g/d									
0–7 d	150.24 ^c^	159.43 ^bc^	212.00 ^ab^	160.86 ^bc^	228.50 ^a^	9.76	0.017	0.847	0.011
8–14 d	252.14	285.93	298.86	307.57	317.79	11.88	0.439	0.079	0.610
0–14 d	201.19 ^b^	222.68 ^ab^	255.43 ^a^	234.21 ^ab^	273.14 ^a^	8.26	0.032	0.005	0.749
ADFI, g/d									
0–7 d	205.36	225.37	266.11	205.09	255.31	10.13	0.180	0.246	0.610
8–14 d	447.38	525.96	495.83	479.94	532.42	17.15	0.512	0.317	0.796
0–14 d	328.57	374.57	380.97	355.54	392.33	11.14	0.387	0.174	0.591
F/G, g/d									
0–7 d	1.37 ^a^	1.43 ^a^	1.26 ^ab^	1.31 ^ab^	1.12 ^b^	0.03	0.027	0.005	0.245
8–14 d	1.82	1.85	1.69	1.56	1.70	0.05	0.290	0.105	0.508
0–14 d	1.64 ^ab^	1.68 ^a^	1.50 ^bc^	1.51 ^bc^	1.44 ^c^	0.03	0.009	0.001	0.865

Note: ADFI = average daily feed intake; ADG = average daily gain; F:G = feed:gain ratio; SEM = standard error of the mean. Within a row, means without a common superscript letter differ at *p* < 0.05.

**Table 3:** The effect of dietary protein level on diarrhea in weaned piglets.

Items	CP Level, %					SEM	*p*-Value		
	14%	15%	16%	17%	18%		ANOVA	Linear	Quadratic
Diarrhea rate, %									
0–7 d	11.43	8.00	7.62	8.57	12.26	0.95	0.42	0.74	0.06
8–14 d	0.95	1.71	0.95	1.14	1.43	0.45	0.99	0.91	1.00
0–14 d	6.19	4.86	4.29	4.86	6.85	0.60	0.65	0.76	0.14
Diarrhea index,									
0–7 d	0.24	0.19	0.17	0.19	0.24	0.02	0.80	1.00	0.22
8–14 d	0.02	0.03	0.02	0.05	0.02	0.01	0.88	0.68	0.64
0–14 d	0.13	0.11	0.09	0.12	0.14	0.05	0.89	0.84	0.35

## Data Availability

The data presented in this study are available on request from the corresponding author (the data are not publicly available due to privacy or ethical restrictions).

## Supplementary Materials

The following supporting information can be downloaded at: https://www.mdpi.com/article/10.3390/ani16162464/s1, Table S1: Raw data of the Venn diagram analysis of serum metabolites in weaned piglets fed diets with 14%, 16%, and 18% crude protein levels; Table S2: Raw data of the hierarchical clustering analysis based on variable importance in projection (VIP) values of serum differential metabolites in weaned piglets; Figure S1: Permutation test (200 permutations) for the PLS-DA model. Blue bars show the frequency distribution of Q^2^ from random permutations, and red bars show permuted R^2^Y.

## References

[1] Guevarra R.B., Lee J.H., Lee S.H., Seok M.J., Kim D.W., Kang B.N., Johnson T.J., Isaacson R.E., Kim H.B. (2019). Piglet gut microbial shifts early in life: Causes and effects. J. Anim. Sci. Biotechnol..

[2] Saladrigas-García M., D’angelo M., Ko H.L., Nolis P., Ramayo-Caldas Y., Folch J.M., Llonch P., Solà-Oriol D., Pérez J.F., Martín-Orúe S.M. (2021). Understanding host-microbiota interactions in the commercial piglet around weaning. Sci. Rep..

[3] Wang R., Hou L., Wu Q., Wen X., Xiong Y., Yang X., Gao K., Jiang Z., Cao S., Wang L. (2026). The Role of CP Level and Interaction with Antibiotics in the Post-Weaning Piglets’ Diet: Growth Performance, Body Composition, Nutrient Digestion, and Intestinal Health. Animals.

[4] Ren Z., Fan H., Deng H., Yao S., Jia G., Zuo Z., Hu Y., Shen L., Ma X., Zhong Z. (2022). Effects of dietary protein level on small intestinal morphology, occludin protein, and bacterial diversity in weaned piglets. Food Sci. Nutr..

[5] Ren Z., Zhang X., Fan H., Yu Y., Yao S., Wang Y., Dong Y., Deng H., Zuo Z., Deng Y. (2023). Effects of different dietary protein levels on intestinal aquaporins in weaned piglets. J. Anim. Physiol. Anim. Nutr..

[6] Tang Q., Lan T., Zhou C., Gao J., Wu L., Wei H., Li W., Tang Z., Tang W., Diao H. (2024). Nutrition strategies to control post-weaning diarrhea of piglets: From the perspective of feeds. Anim. Nutr..

[7] Xia J., Fan H., Yang J., Song T., Pang L., Deng H., Ren Z., Deng J. (2022). Research progress on diarrhoea and its mechanism in weaned piglets fed a high-protein diet. J. Anim. Physiol. Anim. Nutr..

[8] Jansman A.J.M., Cirot O., Corrent E., Lambert W., Ensink J., van Diepen J.T.M. (2019). Interaction and imbalance between indispensable amino acids in young piglets. Animal.

[9] Yu D., Zhu W., Hang S. (2019). Effects of low-protein diet on the intestinal morphology, digestive enzyme activity, blood urea nitrogen, and gut microbiota and metabolites in weaned pigs. Arch. Anim. Nutr..

[10] Luise D., Chalvon-Demersay T., Correa F., Bosi P., Trevisi P. (2023). Review: A systematic review of the effects of functional amino acids on small intestine barrier function and immunity in piglets. Animal.

[11] Fu R., Liang C., Chen D., Tian G., Zheng P., He J., Yu J., Mao X., Gu Z., Yang W. (2023). Effects of low-energy diet supplemented with betaine on growth performance, nutrient digestibility, and serum metabolomic profiles in growing pigs. J. Anim. Sci..

[12] Li Y., Yin J., Han H., Liu G., Deng D., Kim S.W., Wu G., Li T., Yin Y. (2018). Metabolic and Proteomic Responses to Long-Term Protein Restriction in a Pig Model. J. Agric. Food Chem..

[13] Deng D., Yao K., Chu W., Li T., Huang R., Yin Y., Liu Z., Zhang J., Wu G. (2009). Impaired translation initiation activation and reduced protein synthesis in weaned piglets fed a low-protein diet. J. Nutr. Biochem..

[14] Zhou H., Chen D., Mao X., He J., Yu J., Zheng P., Luo J., Gao J., Htoo J.K., Yu B. (2019). Evaluation of standardized ileal digestible lysine requirement for 8–20 kg pigs fed low crude protein diets. Anim. Sci. J..

[15] Li Y., Shi P., Yao K., Lin Q., Wang M., Hou Z., Tang W., Diao H. (2024). Diarrhea induced by insufficient fat absorption in weaned piglets: Causes and nutrition regulation. Anim. Nutr..

[16] Jayaraman B., Nyachoti C.M. (2017). Husbandry practices and gut health outcomes in weaned piglets: A review. Anim. Nutr..

[17] Tang X., Xiong K., Fang R., Li M. (2022). Weaning stress and intestinal health of piglets: A review. Front. Immunol..

[18] Marchetti R., Faeti V., Gallo M., Pindo M., Bochicchio D., Buttazzoni L., Della Casa G. (2023). Protein Content in the Diet Influences Growth and Diarrhea in Weaning Piglets. Animals.

[19] Heo J.M., Opapeju F.O., Pluske J.R., Kim J.C., Hampson D.J., Nyachoti C.M. (2013). Gastrointestinal health and function in weaned pigs: A review of feeding strategies to control post-weaning diarrhoea without using in-feed antimicrobial compounds. J. Anim. Physiol. Anim. Nutr..

[20] An J., Lee J., Song M., Oh H., Chang S., Song D., Cho H., Park S., Jeon K., Kim H.B. (2025). Supplementation of protease with low protein diets improves incidence of frequency and nitrogen metabolism in weaned pigs. J. Anim. Sci. Technol..

[21] Rodrigues L.A., Wellington M.O., González-Vega J.C., Htoo J.K., Van Kessel A.G., Columbus D.A. (2021). Functional amino acid supplementation, regardless of dietary protein content, improves growth performance and immune status of weaned pigs challenged with Salmonella Typhimurium. J. Anim. Sci..

[22] Wellington M.O., Hulshof T.G., Ernst K., Balemans A., Page G.I., Van Hees H.M. (2023). Impact of L-Arginine and L-Glutamine supplementation on growth performance and immune status in weanling pigs challenged with Escherichia coli F4. J. Anim. Sci..

[23] Zhang X., Qiu K., Wang L., Xu D., Yin J. (2018). Integrated Remodeling of Gut-Liver Metabolism Induced by Moderate Protein Restriction Contributes to Improvement of Insulin Sensitivity. Mol. Nutr. Food Res..

[24] Spring S., Premathilake H., DeSilva U., Shili C., Carter S., Pezeshki A. (2020). Low Protein-High Carbohydrate Diets Alter Energy Balance, Gut Microbiota Composition and Blood Metabolomics Profile in Young Pigs. Sci. Rep..

[25] Mandal A., Pal D., Mitra A.K. (2016). Circumvention of P-gp and MRP2 mediated efflux of lopinavir by a histidine based dipeptide prodrug. Int. J. Pharm..

[26] Salaga M., Binienda A., Draczkowski P., Kosson P., Kordek R., Jozwiak K., Fichna J. (2018). Novel peptide inhibitor of dipeptidyl peptidase IV (Tyr-Pro-D-Ala-NH(2)) with anti-inflammatory activity in the mouse models of colitis. Peptides.

[27] Daniel H. (2004). Molecular and integrative physiology of intestinal peptide transport. Annu. Rev. Physiol..

[28] Rahabi M., Salon M., Bruno-Bonnet C., Prat M., Jacquemin G., Benmoussa K., Alaeddine M., Parny M., Bernad J., Bertrand B. (2022). Bioactive fish collagen peptides weaken intestinal inflammation by orienting colonic macrophages phenotype through mannose receptor activation. Eur. J. Nutr..

[29] Cheng J., Wu P., Li C., Han Y., Sun M., Dou Y., Chen S., Zhang J. (2026). Inflammation-triggered self-immolative conjugates enable oral peptide delivery by overcoming gastrointestinal barriers. Sci. Adv..

