# Supplementary material for: Effects of Dietary Crude Protein Level on Growth Performance, Immune Function, and Serum Metabolic Profile in Weaned Piglets
Source: Animals (Basel). 2026 Aug 8;16(16):2464. doi: 10.3390/ani16162464 (PMC13508907; doi:10.3390/ani16162464)
Supplement: Supplementary file 1 [file animals-16-02464-s001.zip › Supplement Figure.pdf]

Effects of Dietary Crude Protein Level on Growth Performance, Immune Function, and Serum Metabolic Profile in Weaned Piglets

Tao Wang[1,2], Jin Chen [1,2], Siyu Xiong [1,2], Xiurong Peng [1,2], Jinyuan Liu [1,2], Dong Qu [1,2], Bin Li [1,2] and Jiabao Yang [1,2,]*

1 Sichuan Animal Science Academy, Chengdu 610066, China
2 Animal Genetic Breeding and Reproduction Key Laboratory of Sichuan Province, Chengdu 610066, China
* Correspondence: 13699007358@163.com

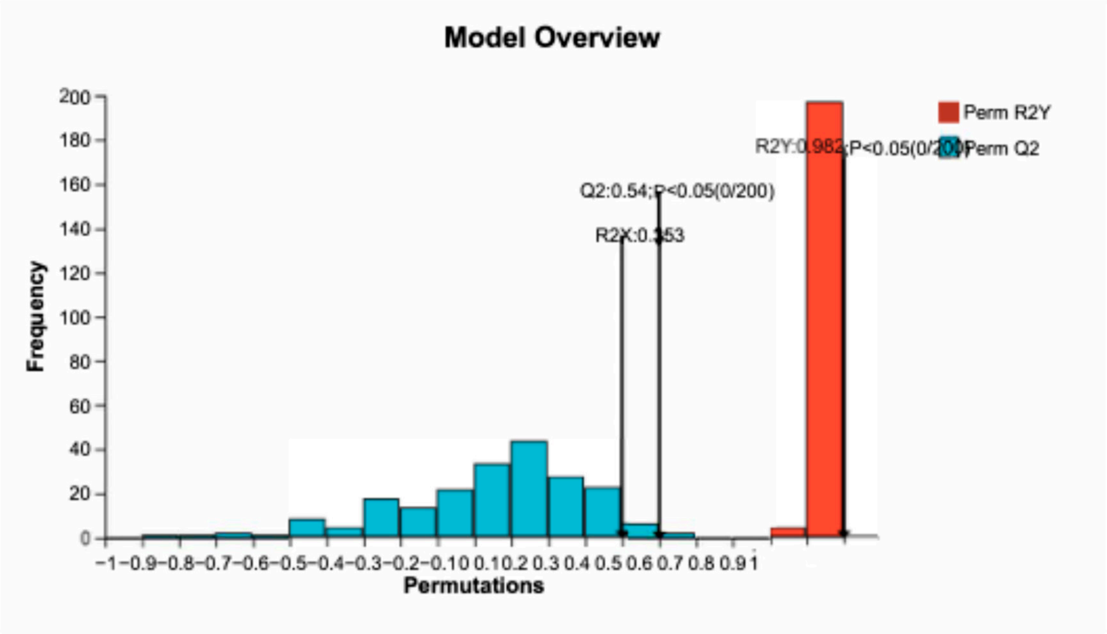

Figure S1. Permutation test (200 permutations) for the PLS-DA model. Blue bars show the frequency distribution of Q^2 from random permutations, and red bars show permuted R^2Y.
